# Young People’s Attitudes and Motivations Toward Social Media and Mobile Apps for Weight Control: Mixed Methods Study

**DOI:** 10.2196/11205

**Published:** 2019-10-10

**Authors:** Charoula Konstantia Nikolaou, Zoey Tay, Jodie Leu, Salome Antonette Rebello, Lisa Te Morenga, Rob M Van Dam, Michael Ernest John Lean

**Affiliations:** 1 Graduate School of Public Health Department of Biostatistics and Bioinformatics St Luke's International University Tokyo Japan; 2 Saw Swee Hock School of Public Health National University of Singapore Singapore Singapore; 3 Research School of Population Health College of Health and Medicine Australian National University Canberra Australia; 4 Department of Human Nutrition University of Otago Dunedin New Zealand; 5 Department of Human Nutrition School of Medicine, Dentistry & Nursing University of Glasgow Glasgow United Kingdom

**Keywords:** weight gain, young adults, obesity, public health, focus groups, mobile apps, mHealth

## Abstract

**Background:**

Effective prevention at a young enough age is critical to halt the obesity epidemic. Mobile health (mHealth) apps would potentially reach large numbers at low-cost. While there is already a profusion of lifestyle apps, they are mostly non-evidence-based and evidently ineffective against rising obesity prevalence.

**Objective:**

The aim of this study was to explore preferences and usage of lifestyle apps among young people in 6 countries.

**Methods:**

A mixed methods study was conducted among young people aged 13 to 24 years residing in the United Kingdom, Belgium, Finland, Greece, Singapore, and New Zealand. Participants were recruited from Web advertisements on Facebook, asking for volunteers interested in mobile apps in general, not specific to lifestyle or health, to complete a short survey comprising 18 questions on demographics, weight gain, and mobile app preferences and then to join English-language online focus groups, which were held during 2017, in password-protected Web rooms, moderated by an experienced researcher. Descriptive statistics were carried out for the survey, and thematic analysis was applied to transcripts.

**Results:**

A total of 2285 young people (610 adolescents aged 13-17 years and 1675 young adults aged 18-24 years) responded and completed the survey, with 72.0% (1645) reported being concerned about weight gain for themselves or friends. Later, 807 young people (376 adolescents and 431 young adults) were selected based on age and country to participate in 12 online focus groups, with 719 young people completing. Analysis revealed 4 main themes: (1) feelings toward personal weight; (2) perception of lifestyle apps and desired content for weight gain prevention; (3) social media apps, lifestyle apps, and motivation for downloading and retaining; and (4) data safety and data usage and confidentiality. Young people are interested in evidence-based advice in programs incorporating their preferences.

**Conclusions:**

Young people are commonly, and consistently across 6 countries, concerned about weight gain and obesity and would welcome evidence-based mHealth programs, provided the views of young people themselves are incorporated in the program content.

## Introduction

### Background

Obesity is one of the biggest public health challenges of the 21st century. At least 650 million adults are currently obese worldwide [1], and effectiveness of obesity treatments is rather modest [2]. Effective prevention methods are needed to halt the obesity epidemic.

Obesity develops with age and can only be prevented before it has developed. The transition from adolescence to young adulthood is a critical life period with rapid weight gain [3-5]. We currently have the largest ever generation of adolescents and young people and the most vulnerable to obesity and secondary noncommunicable diseases in human history [6]. Effective promotion of sustainable health behaviors to prevent obesity must specifically target people where primary prevention is still possible. At age 18 years, the prevalence of body mass index (BMI)>30 kg/m^2^ (5.8%) and BMI>25 kg/m^2^ (22.8%), are still relatively low, which more than double by age 35 years [7]. Body weights rise most rapidly between ages 13 to 24 years internationally [8-14], with steeper weight gain trajectories most likely to reach BMI>30 kg/m^2^, now affecting 40% by age 65 years in the United Kingdom [15].

Adolescence and young adulthood present vital opportunities for intervention, with multiple lifestyle changes for emerging young adults—self-determining, financially independent, outside parental controls [16]. Social interactions often revolve around heavily marketed foods and drinks, tending to promote greater consumption [17]. Young people tend to be relatively neglected by conventional health promotion, considered *hard to reach* through lack of interface with health information dissemination or engagement with health professionals [18]. The internet has become the dominant mode for communication, information exchange, and increasingly for health care delivery. Thus, electronic health (electronic technology in health care) and mobile health (mHealth; mobile technology in health care) offer numerous advantages above traditional methods [19]. Although conventional didactic methods lack reach, innovative electronic learning and support can prevent unwanted weight gain in young adults [20]. Transferring existing online resources with proven effectiveness for weight gain prevention into mobile apps could reach a much greater proportion of the at-risk young population. Near-universal mobile phone ownership, projected to reach 5 billion by 2019, has been matched by increasing availability and access to mHealth apps, mostly for weight loss and fitness [21]. Mobile app downloads reached 149.3 billion in 2016 [22]. Over 25,000 apps are currently aimed solely at weight management [23], but few (0.17%) report having incorporated experts’ and users’ inputs during development. Even fewer have been tested for effectiveness, mostly with poor results [24,25]. Efforts to treat established obesity in young people have been rather ineffective [26,27], and there are no apps specifically tailored to offer weight gain prevention for young people.

### Objectives

This study used newer information technology (IT) methods to explore the views of young people from several countries on weight gain and its prevention and their opinions and preferences to inform development of weight gain prevention apps tailored to the needs of young people.

## Methods

### Overview

A *mixed methods* study design was used, with an online survey followed by online asynchronous focus groups [28]. This approach integrates quantitative and qualitative data within a single investigation, to permit a more complete and synergistic utilization of data than separate quantitative and qualitative data collection and analyses [29,30]. The study was approved by the Institutional Ethical Review Board of the Catholic University of Louvain.

### Questionnaire Design and Recruitment

The recruitment questionnaire comprised 18 questions: 2 multiple choice questions (country of residence, current occupation), 7 dichotomous questions (parents’ academic degree, parents’ smoking, participants’ smoking, concern about weight gain, interest in a quiz to estimate risk of weight gain, current use of mobile apps, interest in an app specifically designed by researchers on weight gain prevention), 8 demographic questions (gender, age range 13-24 years, postcode, current weight, current height, current waist size, dress size, email address), and 1 open-ended question (if you are using lifestyle mobile apps, which ones are you currently using; Multimedia Appendix 1).

The questionnaire was developed following an iterative process, through discussions with all coauthors based in the different countries participating in this study, to ensure that the questions were culturally appropriate. A draft questionnaire was trialed among colleagues first, and after necessary edits, the final questionnaire was trialed with a small group of young people to ensure all functions worked in the online environment and that the wording of the questions was appropriate for this age group, especially for the countries where English is not the official first language.

This was a closed survey, available only to young people aged between 13 to 24 years and residing in 1 of the 6 countries (Belgium, Singapore, New Zealand, Finland, UK, and Greece). Young people were recruited via an online advertisement service (Facebook). The advertisements appeared in a pop-up format, in the profiles of those who declared their age to be between 13 to 24 years and residing in 1 of 6 countries where English is either an official language (United Kingdom, New Zealand, and Singapore) or widely spoken and understood among young people (Greece, Belgium, and Finland). The advertisement invited young people to help guide researchers in building a mobile app, with a link to the online recruitment questionnaire. Participants were not required to have any particular interest in weight management or health, and no financial or other incentives were offered. The recruitment questionnaire was available in 2 screens in total; the first containing the subject information sheet with information on the study and the second containing the questions. The collected data were managed by SurveyMonkey, where they were automatically recorded and saved. When the advertisement and recruitment closed, data were downloaded and transferred to a university computer, which is encrypted to ensure data protection. Internet protocol addresses were recorded automatically when participants completed the online survey and checked to ensure no multiple entries were submitted by the same participant.

**Figure 1:**
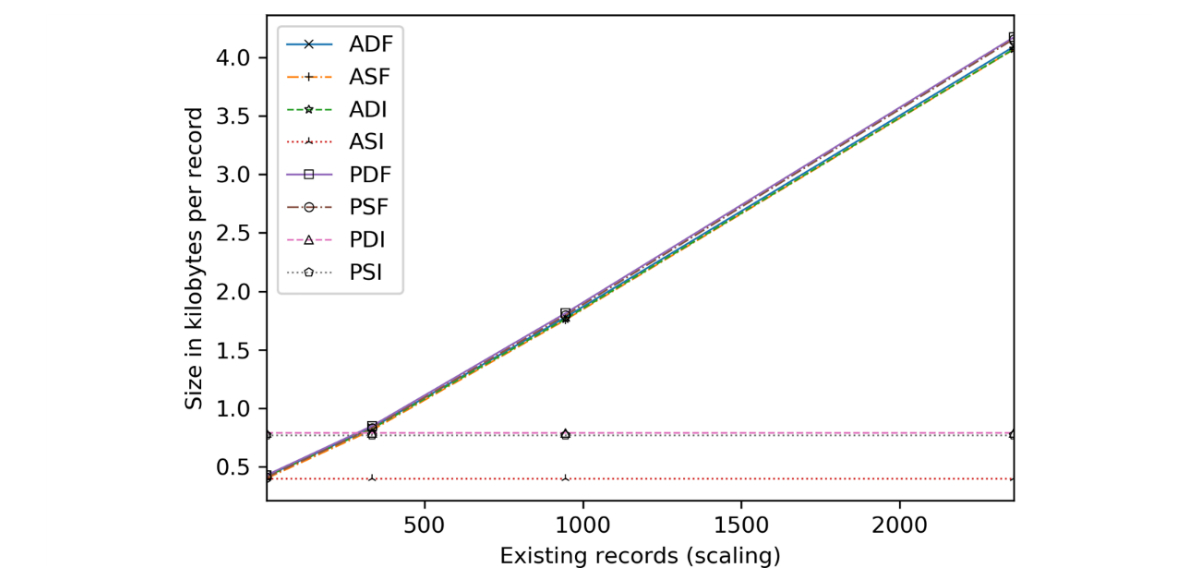
Study procedures for participants completing the survey and those selected to participate in the focus groups. FG: focus group.

A subject information sheet with information on the study and the principal investigator and contact details to express any concerns they might have on the questionnaire was incorporated in the first page of the questionnaire. People following the link could read the information and decide if they wanted to proceed with the questionnaire. No data were stored for incomplete questionnaires. Respondents were assured anonymity but also invited to provide their email address if they wished to participate in an online focus group in which some of the questions could be discussed further. Those who volunteered to participate in the focus groups were first separated by age group into *adolescents* (13-17 years) or *young adults* (18-24 years) and then block-randomized into 8 groups by gender and country, so that these characteristics were uniformly distributed across groups (Figure 1). The focus groups were conducted on an online platform in password-protected Web rooms (ProBoards). The focus groups were asynchronous, open 24 hours/day for 2 weeks to accommodate participants from all different time zones and life/school/work schedules, allowing more views to be documented.

The focus group moderator (CKN) posted information on the study, ground rules on group etiquette, confidentiality, ethical considerations and consent, and a discussion guide in every Web room. A discussion guide (Multimedia Appendix 2) was created to guide the focus groups, with open-ended questions and prompts to introduce preselected topics: (1) current use of lifestyle apps, (2) factors motivating downloading and retaining apps, (3) use of social media, (4) concerns about weight gain, and (5) concerns about environmental and ethical issues around food and drink production and distribution. Topics 3 and 4 (Multimedia Appendix 2) were included based on the results of a previous study in young adults that found 2 different behavioral change theories: the *rational* and the *stealth* theories to be effective in preventing the usual weight gain in this population [20]. The rational choice theory by Simon (1955) [31] states that humans, provided they have the right information, will make a rational choice [31], whereas the stealth theory by Robinson (2010) [32] states that humans can change a behavior if they find a behavior that is motivating in itself [32].

Online focus groups have advantages and disadvantages compared with conventional face-to-face focus groups. They can involve large numbers; an anonymity may permit more open answers: the moderator emphasized that all responses were welcomed, that none would be considered right or wrong, but that they were monitored for any inappropriate content or behavior. Being unable to share facial expressions, which contribute to face-to-face focus groups, participants were encouraged to use emoticons in their postings which were used in the analysis.

### Statistical Analysis

Descriptive statistics used SPSS 24 (IBM). Differences between groups were tested with the MedCalc statistical software (MedCalc). Weight, height, and BMI centiles for adolescents (aged 13-18 years) were calculated according to the International Obesity Task Force [33].

### Qualitative Analysis

Transcribed data were transferred to software (NVivo, version 11, QSR International) for analysis. Thematic analysis was chosen as the most appropriate analytical method through its ability to identify patterned meanings across a dataset and responses [34]. Coding and analysis were carried out using as described by Braun and Clark [35]. Analysis of transcripts started with multiple readings by the principal researcher (CKN) to identify keywords and phrases. Relevant words and phrases identified were coded, and data were categorized describing main issues reported by participants.

All initial codes relevant to the discussion guide and research question were grouped into themes by combining similar codes. Codes and themes were triangulated between the first and last authors to enhance the validity of the coding phrases, and all authors reviewed the selective codes, themes, and exemplar quotes. *Main themes* emerged from relevant combinations of named themes, each accompanied by a detailed analysis, to explain the salient sections of data collected from the focus groups.

### Reflexivity Statement

Reflexivity relates to sensitivity to the ways in which the researcher and the research process may shape the data collected, including the role of prior assumptions and experience. This study was carried out online; hence, the researcher had minimal contact with the participants during recruitment and conducting of the focus groups. The participants could enter the Web room at any time and from any place when they felt comfortable to do so. Our study was designed to elicit contributions from a broad range of participants. During the focus group process, no individual’s views were preferred over those of others. A goal of data analysis was the identification of common themes that emerged from comparison across the focus groups. However, equal importance was attached to the analysis focusing on the details of individuals’ reports relating to specific views and experiences as well as those of the groups as a whole.

## Results

### Overview

Results are reported according to the Checklist for Reporting Results of Internet E-Surveys [36].

Over an 84-hour period after posting the Facebook advertisement, 2285 young people aged 13 to 24 years (610 *adolescents* aged 13-17 years and 1675 *young adults* aged 18-24 years) responded to the advertisement and completed the survey. Demographic information for those completing the survey is presented in Tables 1 and 2.

Young people (1645/2285, 71.99%) reported being concerned about weight gain for themselves or friends, with more young adults (aged 18-24 years) being concerned than adolescents (aged 13-17 years; χ^2^_1_=18.6, *P*<.001). There were no differences between countries among young adults for expressing concern about unwanted weight gain, but there was a greater range for adolescents, for example, between Finland (55.4%) and Greece (76.2%). A similar percentage of young people (1657/2285, 73.30%) reported being interested in a weight gain prevention app, again with significantly more young adults being interested in such an app than adolescents (χ^2^_1_=11.4, *P*=.001). Approximately half of the young adults (49.7%, 833/1676) and one-third of the adolescents (31.0%, 189/610) reported currently using a lifestyle mobile app with significantly more young adults currently using 1 (χ^2^_1_=–172.4, *P*<.001). Among young people, there was no difference in the BMI of those who reported using lifestyle apps and those who did not (21.9 kg/m^2^-22.0 kg/m^2^). Reasons mentioned by participants for not using apps were as follows: time-consuming, not reliable information/source, and too many advertisements. Similarly, for adolescents, there was no significant difference in BMI centiles of those who reported using a lifestyle app and those who did not. In adolescents, reported use of lifestyle apps increased with age, from 27.6% at age 13 years to 40.8% at age 17 years, (χ^2^_1_=23.5, *P*<.001).

**Table 1:** Demographic characteristics of adolescents completing the survey and those selected to participate in the 6 online focus groups.

Demographic characteristics		Survey (n=610)	Focus group 1 (n=64)	Focus group 2 (n=62)	Focus group 3 (n=64)	Focus group 4 (n=62)	Focus group 5 (n=62)	Focus group 6 (n=62)
Age (years), mean (SD)		15.3 (1.4)	15.0 (1.4)	15.0 (1.4)	15.0 (1.4)	15.0 (1.4)	15.0 (1.4)	15.0 (1.4)
Gender (female), n (%)		429 (70.3)	36 (57)	35 (57)	36 (57)	40 (65)	42 (67)	40 (65)
Weight (kg), mean (SD)		59.7 (8.0)	58.2 (7.9)	60.9 (8.3)	57.9 (7.0)	59.5 (9.8)	60.7 (8.2)	59.03 (6.6)
Height (m), mean (SD)		1.64 (0.07)	1.64 (0.07)	1.64 (0.07)	1.63 (0.06)	1.63 (0.07)	1.64 (0.06)	1.64 (0.07)
BMI^a^ (kg/m^2^), mean (SD)		22.2 (8.0)	21.4 (1.9)	22.4 (2.07)	21.6 (1.8)	22.02 (2.4)	22.4 (2.2)	21.9 (1.8)
**Country, n (%)**								
	Greece	97 (15.9)	11 (17)	11 (17)	10 (15)	10 (15)	11 (17)	10 (16)
	Singapore	93 (15.2)	11 (17)	11 (17)	11 (17)	11 (17)	9 (15)	8 (14)
	Finland	74 (12.1)	11 (17)	11 (17)	10 (15)	10 (15)	9 (15)	10 (16)
	United Kingdom	163 (26.7)	11 (17)	11 (17)	11 (17)	11 (17)	11 (17)	12 (18)
	Belgium	105 (17.2)	11 (17)	11 (17)	11 (17)	11 (17)	11 (17)	12 (18)
	New Zealand	78 (12.8)	9 (13)	7 (13)	11 (17)	9 (15)	11 (17)	10 (16)
Parents’ higher education degree (yes), n (%)		433 (71.0)	46 (72)	45 (72)	43 (67)	38 (61)	45 (72)	52 (83)
Parents smoking (yes), n (%)		152 (24.9)	18 (27)	17 (27)	13 (20)	20 (31)	17 (27)	18 (29)
Current use of lifestyle apps (yes), n (%)		189 (31.0)	23 (36)	22 (36)	23 (36)	17 (28)	20 (32)	29 (47)

^a^BMI: body mass index.

**Table 2:** Demographic characteristics of young adults completing the survey and those selected to participate in the focus groups.

Demographic characteristics		Survey (n=1675)	Focus group 1 (n=72)	Focus group 2 (n=73)	Focus group 3 (n=71)	Focus group 4 (n=70)	Focus group 5 (n=73)	Focus group 6 (n=72)
Age (years), mean (SD)		19.7 (1.7)	20.7(1.9)	20.7(1.9)	20.8 (1.9)	20.7 (1.9)	20.7 (1.9)	20.6 (1.9)
Gender (female), n (%)		1170 (69.9)	43 (60)	42 (58)	40 (56)	39 (55)	43 (60)	42 (58)
Weight (kg), mean (SD)		63.8 (11.3)	64.5 (10.2)	63.2 (10.3)	63.5 (10.5)	64.3 (13.5)	62.6 (10.4)	64.2 (10.8)
Height (m), mean (SD)		1.80 (4.3)	1.72 (0.08)	1.71 (0.08)	1.70 (0.09)	1.70 (0.1)	1.70 (0.1)	1.70 (0.07)
BMI^a^ (kg/m^2^, mean (SD)		21.9 (3.2)	21.6 (2.6)	21.5 (2.8)	21.7 (2.9)	22.0 (3.9)	21.6 92.4)	21.9 (3.0)
**Country, n (%)**								
	Greece	316 (18.86)	10 (14)	11 (15)	11 (16)	11 (16)	12 (16)	12 (16)
	Singapore	270 (16.11)	10 (14)	10 (14)	9 (14)	10 (15)	10 (14)	11 (15)
	Finland	211 (12.59)	13 (17)	14 (18)	13 (17)	11 (16)	12 (16)	12 (16)
	United Kingdom	420 (25.07)	14 (18)	14 (18)	14 (18)	15 (19)	14 (18)	14 (19)
	Belgium	219 (13.07)	12 (16)	11 (15)	11 (16)	10 (15)	13 (17)	11 (15)
	New Zealand	239 (14.26)	13 (17)	13 (17)	13 (17)	13 (17)	12 (16)	12 (16)
**Occupation, n (%)**								
	In higher education	767 (45.79)	31 (43)	41 (56)	32 (45)	28 (39)	34 (47)	39 (54)
	Employed	670 (40.00)	31 (43)	26 (35)	28 (40)	32 (45)	28 (37)	28 (38)
	Looking for job/other	238 (14.20)	10 (13)	6 (7)	11 (14)	10 (15)	11 (14)	5 (6)
Parents’ higher education degree (yes), n (%)		1280 (76.41)	59 (82)	55 (75)	53 (75)	48 (68)	56 (77)	57 (79)
Parents’ smoking (yes), n (%)		390 (23.28)	17 (23)	12 (17)	12.2 (17)	14 (20)	16 (21)	19 (26)
Smoking (yes), n (%)		273 (16.29)	15 (20)	13 (18)	10 (14)	12 (17)	12 (16)	14.2 (18)
Current use of lifestyle apps (yes), n (%)		832 (49.67)	34 (47)	35 (48)	44 (62)	40 (57)	31.5 (43)	41 (56)

^a^BMI: body mass index.

From the 2285-young people who completed the survey, 807 young people (376 adolescents aged 13-17 years and 431 young adults aged 18-24 years) offered and were contacted to participate in the focus groups, stratified by country and age. A total of 12 focus group sessions, held in total at the start of 2017, were completed by 719 young people (completion rate 89%). Detailed demographic characteristics of focus group participants are presented in Tables 1 and 2. Due to the large number of participants, some quantitative analysis was possible. The mean number of individual entries into each focus group for the adolescents was 98 (SD 6.0) and for the young adults 121 (SD 12.0). The moderator tried to observe rather than participate to allow participants to freely express themselves. The moderator checked the Web rooms for any inappropriate content every 12 hours and interacted individually with a total of 23 participants. Moreover, because of the nature of the asynchronous groups and participants being based in 6 countries, all but 2 (Belgium and Finland) in different time zones, participants and moderator logged in at different times and they were not online concurrently.

### Main Themes

Main themes emerging from both adolescents’ and young adults’ focus groups are identified and presented with a general explanation of findings and exemplar quotes. Some of the main themes emerging, unsurprisingly, matched the topics which had been introduced into the focus groups.

#### Theme 1: Feelings Toward Personal Weight

Young people in all countries reported that weight changes are of concern for them (568/719, 78.9% of participants). Although most participants were neither overweight nor obese, they considered body weight an important indicator of health and fitness. To the adolescent participants, weight changes were considered an important part of the *growing-up* process, but they questioned what is considered *normal* weight gain. Adolescents also expressed concerns about the best way to voice their issues around body weight, diet, and physical activity to parents and friends, with difficulties making lifestyle choices free from families’ or friends’ influences and used emoticons expressing negative feelings, for example, sad face, face crying, for unwanted weight gain:

I think I am really fat because previous cloths don't fit me. And I think I am going to get health problems according to my weight. I do exercise regularly. It would be awesome to have some info on what is normal weight.

Male, 14 years, Finland

I'm one of the tallest girls in the year, and scared one of the heaviest as well, people keep saying it's because of your height but is height the only reason or should I be doing more things? I don’t think I eat that much and I already do gym 5 hours a week but is this not enough maybe?

Female, 13 years, New Zealand

Young adults discussed how weight changes related to taking full responsibility for their lifestyles, while the other significant changes in their lives were occurring. Factors identified for weight increase were types of food available, food costs, and promotion of unhealthy foods such as crisps and chocolate. Increased alcohol consumption was another reason identified for weight gain, particularly by young adults in the United Kingdom and New Zealand, invoking use of emoticons, mostly of sad face:

I put on a lot of weight. Clothes do not fit me. I tried with my friends to lose weight with things like going for a run but then we would go out drinking so...didn’t lose any weight in the end.

Male, 22 years, New Zealand

#### Theme 2: Perceptions of Lifestyle Apps and Desired Content of a Weight-Gain Prevention App

Most participants (546/719, 75.9%) said that they would be interested in app access to electronic information on weight gain prevention. All young people reported being already exposed to abundant information on different diets, *super foods*, and physical activity regimes through websites, magazines, or articles shared through social media. This plethora of information, often conflicting, was difficult to process, and they would welcome advice and information from a source they could trust. Young people again used emoticons to illustrate these feelings, of face confused, face spinning, and sad face. Having access to trustworthy and evidence-based information from what they consider a reliable source was very commonly mentioned (n=489, 68.0% of participants). They mistrust and cannot connect with the general guidelines provided by government agencies but think that information coming from independent researchers and health care professionals would be trustworthy:

You read all this news like eat chocolate because it helps with depression one day and then the other don’t because it has a lot of sugar. Not sure what to believe. Someone like a doctor needs to filter this information.

Female, 14 years, Greece

There is so much info on diets, nutrition, exercise out there. It feels like constantly being bombarded by so many things and in school you are being told something else and at home something else. Those people doing research seem to know things better. Maybe they could give monthly updates on these topics with accurate information.

Female, 16 years, Belgium

On the basis of previous research on online resources for young people to prevent unwanted weight gain, participants were asked to discuss what type of information should be included in a weight gain prevention app. Examples and prompts from the previous study were provided [20]. Most identified diet as the most important factor for weight changes. They wanted information on what is good to eat without promoting excessive weight gain, particularly snacks, and what are *normal* weight gain trajectories.

When asked about environmental and commercial issues around food, they expressed concern over whether chemical or antibiotics had been used and whether people are forced to work in certain food production sectors. These issues/concerns were reported mostly from young people older than 16 years. Participants also discussed wider geopolitical aspects of food practices, especially how corporations producing and handling food affect consumers, society, and the environment including climate change. Adolescents in all countries reported having school lessons on environmental issues, associating certain food habits with environmental issues and current social movements. They wanted better information over how food choices affect the planet and felt it should be online to better inform decisions and actions for more people. They proposed either joining existing social movements, or creating new ones, according to local needs and characteristics:

I read about the food miles. It is crazy when we have tomatoes or apples produced here to buy from other countries. I don’t understand why. This can’t be good for anyone.

Male, 17 years, Greece

I read in the news often about people working in the fish boats like slaves. I don't want to consume the product of this trade.

Male, 19 years, New Zealand

There is a community garden close to my home, and sometimes I go with my dad there. I think it is a great idea to use this land to get local food. A lot better than buying food from the supermarket that travelled from the other side of the world.

Female, 18 years, United Kingdom

#### Theme 3: Social Media Apps, Lifestyle Apps, and Motivation for Downloading and Retaining

Young people reported using some kind of app every day, mostly those linked to social media and instant communication (719/719, 100% of participants), the most popular cited being Facebook, Instagram, Twitter, and Snapchat. This finding was similar across different countries. Young people reported using Facebook mostly for its messaging service or in closed groups for school work. Instagram is very popular because it is considered more private and less commercialized than other social media:

Facebook is kind of dead. Everybody used to have it and most of us will still do but not really for posting anything.

Male, 16 years, Belgium

Instagram hasn't been flooded with the older generation yet (not everyone has an Instagram), it’s “hip” and “cool.” There are no links.

Female, 17 years, New Zealand

Young people reported using Twitter for following news and people they are interested in but not for posting new content of their own as they feel it is less private. Snapchat was felt to carry a lot less social pressure, allowing young people to be more authentic, and to be both addictive and liberating. They felt closed groups on Facebook were most suitable for sharing longer pieces of information on weight gain prevention, and Snapchat or Twitter for short messages.

Young people were not aware of the entire range of lifestyle apps (diet, physical activity) currently available on the market. When they reported using lifestyle apps, these were either 1 of the most popular apps advertised in app shops or had been recommended by friends or family. Young people reported using those for weight management and particularly for increasing/monitoring their physical activity. Some reported using the default lifestyle apps incorporated into their mobile phone device. Poor retention was frequent for lifestyle apps: the main reasons for discontinuing were taking too much time to use them and find the information they wanted and receiving too frequent and too general reminders, which participants found annoying:

I thought I wanted to have a calorie input thing, so that I can put how many calories I had that day but it took too much time and got bored of it soon.

Female, 23 years, Singapore

Finding the right motivation to exercise and eat healthily is also important for young adults:

I'm quite lazy and need motivation to exercise and be fit. When living at home, my mum always reminded me of exercising and cooked nice meals. Would really love if someone would message me and maybe motivate each other online?

Male, 19 years, Belgium

#### Theme 4: Data Safety and Usage, Confidentiality

During the discussion on what technical features young people would like in a lifestyle app, the issues of safety, data usage, and confidentiality emerged most frequently. They were particularly concerned about releasing personal details or allowing apps to have access to personal data or other accounts (emails, Facebook, etc) on mobile phones. They were aware of their *digital footprints*. They did not wish to post traceable personal data or opinions, as they may change their mind later, and such posts might disadvantage them in the future. Online reputation and identity seem to be a very important discussion point among young people (438, 61.0% of participants). However, they would value some form of support to encourage them to continue using an app, for example, *chat bots*, and they were happy to engage with their peers via anonymous online chats or direct SMS (short message service) text messaging:

Your tweets are easily searchable on Twitter which is ok but not good if you want to be yourself.

Male, 20 years, United Kingdom

There are some apps that collect a lot of your personal data. They ask for access to your contacts, photos etc. I have never thought about it previously but why an app needs to have access to my personal stuff?

Male, 18 years, New Zealand

I had apps downloaded that had all of these ads on random things, some quite inappropriate. I don’t want to view all these adds.

Female, 16 years, Finland

Data usage with mobile apps was another important issue. Most young people are on restricted budgets with limited mobile data. Despite the high internet coverage, some young people reported not having internet at home because of cost. It is of great importance to them to avoid draining their battery or mobile data:

Some apps just take up all my data. Maybe have something like a light version like the one Facebook has, the messenger lite.

Female, 17 years, Greece

## Discussion

### Principal Findings

Our results show that young people from 6 different countries are not only concerned about weight changes and diet but also about the relationships with the environment they live in. They would like independent advice to help them negotiate the environment to control weight changes, with lifestyle choices, which also help protect the environment. They are clear that guidance should be in a format that is familiar to them and easily accessible, that is, through a mobile device as long as their concerns are being addressed.

To the best of our knowledge, this is the first study to be conducted among young people at a multicounty level on the potential of mobile apps for weight gain prevention. The mixed online methods used made this possible, bringing young people from 6 countries to the same *room* to discuss topics of common interest. It offered some advantages including the opportunity for participants to provide candid honest, opinions from a position of anonymity. Online focus groups permit many more participants to join and avoid the effort and cost of transcribing recorded discussions, but the resulting discussions may be narrower than in conventional one-to-one focus groups where participants may be more inclined to pick up collateral or tangential issues, and there is a greater opportunity to identify cross-cutting themes. The electronic discussion among young people from different countries set up a stimulus-response reaction that revealed young people’s preferences, concerns, and barriers to using technology for managing their lifestyle. The participants, from 6 countries, reported experiencing our globalized world in a remarkably similar way, sharing common concerns and preferences. Although commonly concerned about unwanted weight gain and welcoming advice on prevention, they are concerned to have reliable information sources and for their data and identities to be protected. Younger people are reliant on mobile communications. They are well adapted to the wider digital environment and also well aware of its potential perils and how their online activity might affect their future. As experienced users, they want end products to meet closely their requirements and needs and not waste time or money.

The main topics/themes incorporated in the design of our questionnaire, and in leading the focus group discussions, were chosen because they are already established as frequent areas of concern to young people in other contexts. Other topics, such as online safety, data usage, and professional and trustworthy advice arose in the focus group discussions. It was important for this study to confirm that the main known issues apply to the subset of young people who have body weight concerns and who may be attracted to use apps directed at preventing weight gain. These individuals may have subtly different attitudes toward decision making, health risks, and risks in general, and they are known to be tempted to spend large amounts of money online for products without evidence for value. The results of the questionnaires tend to confirm that young people do indeed have similar areas of concerns around online/app approaches directed at body weight control.

### Comparison With Prior Work

There is growing interest in Web-based approaches for health under the current World Health Organization Research Agenda, with a view to develop *Health in your Pocket* interventions [37]. The European Commission echoes this with calls for a digital single market [38]. Obesity has become a global public health issue, affecting both developed and developing countries. Solutions that have the potential to be scaled up to reach large populations are therefore vital.

Mobile apps seem to have the potential to increase motivation to eat a more healthful diet [39], but limited research is available about the use of mobile apps for health promotion for adolescents [40] and young people. The digitalized world, which younger generations are in tune with, offers ways to implement solutions that are affordable and have global reach [41]. By the year 2020, an entire generation will have grown up in a fully digital world, with computers, internet, mobile phones, and social networking all being second nature.

#### Data Safety/Reliable Independent Information

About one-third of adolescents and half the young adults reported currently using lifestyle apps. This agrees with a previous study where lifestyle apps were reported to be used by 27.6% of adolescents [42]. Those currently using lifestyle apps mostly use either the most popular apps available in app stores or those recommended by close friends, family, or a trustworthy source rather than government agencies. Young people are especially concerned about their data confidentiality and online safety, with good reason. In a survey conducted in the United Kingdom for Safe Internet Day in 2016, it was showed that in the previous year, 82% of teenagers saw hateful content unintentionally on the internet [43]. Privacy policies incorporated into mobile apps targeting young people rarely adapt their policies to the language style or reading level of young people [44].

#### Environmental Protection/Commercial Exploitation/Ethics

Finding the right motivation to engage with weight gain prevention tools is critical for effectiveness. A recent study found that introducing *rewards* provided good motivation to use a dietary assessment app [45]. Another study using young adults’ focus groups found that the use of social media and mobile gaming is an acceptable method for increasing vegetable consumption [46]. Aligning messages to the topical concerns of young people may strengthen the motivation for behavior change: a recent survey conducted by United Nations Children’s Fund and Eurochild found that 41% of the 15,000 young people participating were worried about climate change [47]. This is in agreement with our results, showing that young people are worried about the effect that their food choices may have on the planet and on people involved in food production.

These sentiments suggest promoting *Food Citizenship*: “the practice of engaging in food-related behaviors that support, rather than threaten, the development of a democratic, socially and economically just, and environmentally sustainable food system” [48].

Mobile services and devices have had a sudden boom that allowed very little time for involving the end users in the product designs. Many lifestyle apps are available, but few are evidence-based or had input from the end users in the design process, which may limit success and effectiveness [49,50]. Involving young people in the design process, piloting, and marketing creates a sense of *ownership*, promoting engagement and motivation for using the app [51]. Given the enormous number of current unreliable and non-evidence-based lifestyle apps in the market, it would be reasonable to try first to transfer the already tested and validated resources into mobile formats. Our study provides evidence that young people do want tailored apps, and the insights from online focus groups can inform appropriate app development to meet the needs and preferences of young people as reported by them. Specifically, the process for creating a user-friendly and engaging mobile app on weight gain prevention needs to address safety, cost, online reputation, and the content desired by young people.

### Strengths and Limitations

Although the issues appear very similar in the 6 countries studied, there are limitations. In this study, only English language was used for the online focus groups. The countries were selected as those where large proportions of young people speak English at a high standard, but the participants may have been individuals with relatively high educational attainment, and their views are not necessarily reflective of non-English speakers in those countries. To take account of this issue, we offered the recruitment advertisement in both English and the local languages. The high consistency in results between countries where English is usually the first language across social and educational classes (United Kingdom, Singapore, New Zealand) and those where English is usually a second language (Belgium, Greece, Finland) suggests that the results may be widely generalizable. We did not include developing countries where socially determined obesity risks and diets are very different, and mobile phone ownership is lower. This study used a relatively new recruitment method for research, with advertisements posted on social media. This ensured maximum visibility across the participating countries and unified adoption with the same approach in all countries. The online method allowed for far more young people to participate than traditional focus groups. The advertisements did not mention weight or health to reduce selection bias and to attract a broad range of participants. Both recruitment and conducting methods were low-cost, convenient, and confidential, suited to reaching young people and for addressing sensitive topics such as body weight. There was no requirement for labor-intensive transcription with online focus groups.

The mixed methods design used in this study also helped with gauging this new topic in a more efficient and comprehensive way, by combing qualitative and quantitative data.

All focus group methods engage with subsectors of the target population that are interested in the topic and have time available. The online approach allows a wide section to participate, in their own time, but does introduce some limitations. The lack of face-to-face interaction and visual cues to influence discussions has been mentioned, but freedom from shyness allows participants to exchange views more freely and avoids intimidation. Furthermore, participants used emoticons to express their feelings. Young people are especially familiar with the use of emoticons, and they feel that emoticons can clarify meaning to the text [52]. Our focus groups were mixed in gender and stratified by age and country. Some researchers have preferred gender-specific focus groups for fear of males dominating the discussion—the *peacock effect* [53]. This effect is less likely in the online environment where participants could log in and express views at any time without threat from others. Online focus groups inevitably have some different characteristics from conventional face-to-face focus groups, being akin to moderated discussion fora, or blogs, and whether they generate the same research information needs separate investigation.

### Conclusions

Young people are commonly, and consistently across 6 countries, concerned about weight gain and obesity. Applying no industry evidence-based IT programs to guide young people toward preventing weight gain would be well received, provided that the views of young people themselves are incorporated in the program content and app design.

## Appendix

Multimedia Appendix 1 Recruitment questionnaire.

Multimedia Appendix 2 Discussion guide for the groups.

## Abbreviations

BMI: body mass index
IT: information technology
mHealth: mobile health
