# Supplementary material for: Young People’s Attitudes and Motivations Toward Social Media and Mobile Apps for Weight Control: Mixed Methods Study
Source: JMIR Mhealth Uhealth. 2019 Oct 10;7(10):e11205. doi: 10.2196/11205 (PMC6913715; doi:10.2196/11205)
Supplement: Multimedia Appendix 1 [file mhealth_v7i10e11205_app1.pdf]

- **We are a team of university researchers who want to help young people avoid unwanted weight gain.**
- **We would like your help with this.**
- **We have no connections to any related commercial company or products.**

1. Do you live in

- ◯ Finland
- ◯ Singapore
- ◯ Greece
- ◯ Belgium
- ◯ UK
- ◯ New Zealand

2. Are you

- ◯ Male
- ◯ Female

3. What is your age?

[ ]

4. Are you currently?

- ☐ In school
- ☐ In higher education (college, university, polytechnic)
- ☐ Employed-part time
- ☐ Employed-Full time
- ☐ Looking for a job

5. Does any of your parents have a University degree?

- ◯ Yes
- ◯ No

6. Does any of your parents smoke?

- ◯ Yes
- ◯ No

7. Do you currently smoke cigarettes, or not?

- ◯ Yes, I do
- ◯ No, I do not

8. What is your postcode?

[ ]

9. Are you concerned about weight-gain for yourself or your friends?

- ◯ Yes
- ◯ No

10. Would you be interested in a quiz showing how likely you are to gain unwanted weight in the 5-10 years?

- ◯ Yes
- ◯ No

Other (please specify)

[ ]

11. Do you use any mobile apps on food/diet or physical activity?

- ◯ Yes
- ◯ No

12. What mobile apps related to food/diet or physical activity do you use?

[ ]

13. Would you be interested in an app on food/exercise developed specifically for your age group with scientific information provided by a team of researchers?

- ◯ Yes
- ◯ No
- ◯ Not sure

Other (please specify)

[ ]

14. What is your current weight?

[ ]

15. What is your height?

[ ]

16. What is your waist size?

[ ]

17. What is your dress size?

[ ]

18. Can we contact you to clarify any of the answers above?
Please provide us with your email address.

[ ]
