# Supplementary material for: Young People’s Attitudes and Motivations Toward Social Media and Mobile Apps for Weight Control: Mixed Methods Study
Source: JMIR Mhealth Uhealth. 2019 Oct 10;7(10):e11205. doi: 10.2196/11205 (PMC6913715; doi:10.2196/11205)
Supplement: Multimedia Appendix 2 [file mhealth_v7i10e11205_app2.pdf]

Appendix 2

Interview Guide- Apps for Lifestyle

**Topics/Questions**	**Probes**
**Topic 1: SOCIAL MEDIA ENCOUNTERS RELATED TO FOOD & ACTIVTITY**	
Typically, what do you use social media tools such as, FB, Twitter, Whatsapp, Instagram, net blogs for? Why?<br>- For those who do not use social media tools, why do they not use them?	*E.g. Instagram to look at food and see what friends are doing*
**Q1.** Does what you see in these spaces help you make decisions related to lifestyle choices such as food or physical activity?<br>- If so, how? (For example via advice blogs, facebook news, and advertisments)	Could you describe specific examples?<br>Do these encounters influence your everyday decisions (what or where to eat) or occasional decisions (example which are the cool restaurants to try over the weekend), or both?
**Q2.** There have been advertisments with regards to lifestyle products (for instance packaged foods, restaurants or fitness devices, gym memberships, active holidays, sport events) on social media. Have you noticed them?<br><br>**Q3.** If participants shared experience of being influence by friends' social media post and not advertisements or vice versa,<br>- Why?	Have you made any changes after reading an advert or a post on diet/lifestyle?

Topic 2: Perceptions of food and activity environment	
**Q1.** What do you think a healthier/healthy food choice is? What was your experience like in making such choices?	What makes it easy or difficult?<br><br>Role of school<br><br>Role of environmental factors (availability, accessibility , affordability and taste of healthier vs less healthier options)<br><br>Strategies adopted to make wise food choices<br><br>*When would you not choose an option based on the calories? What range would you pick foods based on it?*
**Q2.** When you eat meals which are not home-cooked, how far would you typically travel to buy the meal?	Near school, or during transit<br><br>What do they consider their "food environment"<br><br>What kind of places do you usually buy food from?<br><br>How much are these items?<br><br>Why do you buy them? Do you like the food you buy?<br><br>How far are you willing to walk? Is it due to time pressure/weather etc?<br><br>What else is available in the vicinity?<br><br>Are the available foods healthy from your perspective?
**Q3.** What do you think being physically active means? What was your experience like in staying physically active?	What makes it easy or difficult?<br><br>(Absence of regular physical activity classes, spending time on computer/ screens, living on campus, peer pressure, built environment)<br><br>Strategies adopted to be physically active
**Topic 3: RATIONAL/OVERT MODEL**	
**Q1.** What do you feel about your current lifestyle choices?	Eating patterns? Physical activity levels?
**Q2.** If you mentioned that you would like to eat more healthfully and/or be more physically active,<br><br>- Why do you want to make these changes?	For weight control, for fitness and/or attractiveness

- What benefits do you think making these food or activity changes will bring you?	

Topic 4: STEALTH MODEL<br>(a) Environmental impacts,<br>(b) Ethical aspects of food production and industry, and<br>(c) Issues around restriction of independence, by commercial controlling or manipulating young people's food availability and food choices.)	
**Q1.** What are your thoughts on big food companies? Do you think big food companies can influence how we eat?	Example of big food companies (Coca-Cola, McDonalds)<br>How we eat: includes what we eat, how much we eat, where we eat and how frequently we eat).<br>Are you aware of the marketing strategies used by big food companies for promoting their foods?<br>**How concerned are you about this? Is this an issue in your view? Big/small issue?**
**Q2.** "Food choices we make can have an impact on our environment." What do you think of this statement?<br>*e.g. fish grown in cramped conditions that are unnatural*	Carbon food-print with non-local foods<br>Food waste<br>**How concerned are you about this? Is this an issue in your view? Big/small issue?**
**Q3.** Do you think that there are ethical or moral issues around how food is manufactured?<br>*e.g. kidnapped fishermen forced to fish to supply fish for world demand*	Fair-trade food<br>Animal farms<br>Concerns around working and human rights and of people working in food production?<br>How concerned are you about this? Is this a small problem in your view?
**Topic 5: Physical activity and diet changes while attending their education institutions**	
**Q1.** What changes have you noticed in your activity patterns since starting school/university/job	More or less?<br>Happy or unhappy?
**Q2.** What changes have you noticed in your eating behaviours since starting school/University/job	Changes in where, how much, how frequently, with whom, and what

<table>
<tr><td colspan="2">Topic 6: Perceptions related to weight gain during NUS or Republic Polytechnic</td></tr>
<tr><td>Q1. What do you think of your weight since starting school/university/job</td><td>How do you feel about it?</td></tr>
<tr><td colspan="2">Topic 7: Current use of Lifestyle Apps
We will now focus on life style apps. These are apps related to food, physical activity and sleep</td></tr>
<tr><td>Q1. Have you had any experience in using lifestyle apps?
- If yes, what has motivated you to download it?
- If no, why?

Q2. Are you still using the apps?
- If yes, why?
- If no, why?

-GPS, location tracking, any privacy concerns about this?

After the app is not free/campaign is over, do they continue the activity?

Any behaviour change before and after the usage of the app?

What if incentives are for social causes? E.g. running a certain amount means donating money to a charity

Apps with cloud storage, aware of privacy issues?

What functions will be good for a lifestyle app?

Home/food delivery app and healthier options?

Chat function: chatbot to ask about health?

WRAP UP
If you can choose one thing to be in a lifestyle app, what would it be?</td><td>What are the current lifestyle apps that you are using?

How do you hear about apps to download? Advice from family, friends? Adverts?

What characteristics and features of apps that attracted you to download it?
What is the longest duration you have used the app for?

What were the characteristics and features of apps that kept you using the apps?

How important is

▪ Monitoring
▪ GPS tracking to provide healthier eating options and opportunities for activity
▪ Personalized feedback
▪ having a companion website
▪ social media links
▪ App log in process
▪ Games features
▪ Rewards?
▪ Chat function?</td></tr>
</table>

Pictures for Topic 4

There are some interesting snack-tricks.

Commercial snacks often can contain a lot of salt, which makes you thirsty. Thirsty people then take in extra calories from sugary drinks or alcohol..... No mystery then that Pepsico now owns Walkers crisps!

Commercial snacks encourage addictive eating or grazing patterns. You may recognize how their marketing promotes external 'cues' to snacking, like meeting friends, having coffee, finishing lectures.

Coca-cola.....?

Kellogg's, world's biggest cereal-maker recently announced that its recent acquisition of the snack 'Pringles' was enough to boost its net income and sales by 15%. Kellogg's appetite for salty snacks is helping fatten its profits!

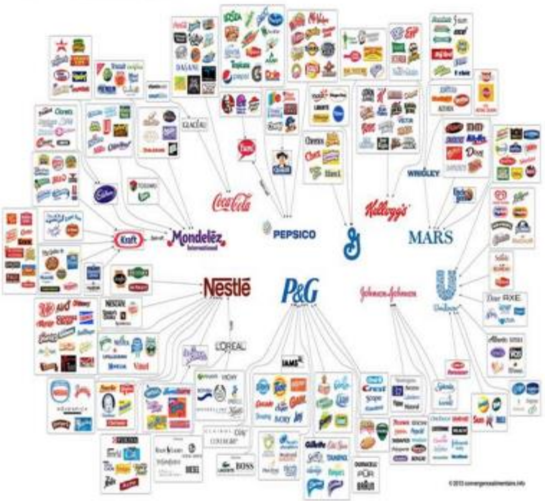

Once upon a time, Christmas was a mid-winter celebration, to call for longer days again.

Families got together, exchanged (cheap) token gifts, and hair was let down during a period with limited light, food and fun.

Santa was dressed in green, to symbolise the green fuse of life. Stockings contained a walnut and a tangerine, Christmas cake or pudding contained beans as tokens of dormant life ('Favours', in cakes, from fava, latin for a bean)

Now, all is red and sparkling. **We have come to believe that without spending, meaningless- gift-giving, excessive drinking and eating, it's not Christmas.**

Supermarkets launched Christmas campaigns in November in attempts to target our emotions and make us buy more....**using marketing lines which people are comfortable with because they resonate with folk-sayings:**

"A little of what you fancy will do you no harm"
"All things are all right in moderation"
"Temptation is there for a good reason"
"Time to cast caution to the wind"
"Just this once..."

Coca-Cola has one of the most successful Christmas campaigns, worldwide.

It is not Christmas until the Coca Cola advert starts!

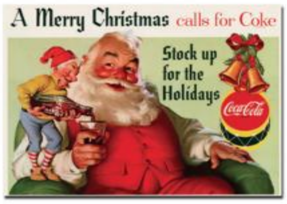

Coca-Cola launched this campaign back in 1930s to increase winter sales when weather was cold.

No joke, the fat, jolly guy with the red suit and cap and rosy cheeks - is the spawn of an advertising campaign by Coca-Cola.

Before then, and still in some less Coca-Colanised countries like Sweden Santa is dressed in Green.

Coca Cola if not solely responsible was heavily instrumental in switching Green Santa to RED. People should have seen RED and STOPPED.
